# Synergistic effect of plasma-activated medium and novel indirubin derivatives on human skin cancer cells by activation of the AhR pathway

**DOI:** 10.1038/s41598-022-06523-x

**Published:** 2022-02-15

**Authors:** Henrike Rebl, Marie Sawade, Martin Hein, Claudia Bergemann, Manuela Wende, Michael Lalk, Peter Langer, Steffen Emmert, Barbara Nebe

**Affiliations:** 1 Department of Cell Biology, Rostock University Medical Center, 18057 Rostock, Germany; 2 Institute for Chemistry, University of Rostock, 18059 Rostock, Germany; 3 Institute for Biochemistry, University of Greifswald, 17487 Greifswald, Germany; 4 Clinic and Polyclinic for Dermatology and Venerology, Rostock University Medical Center, 18057 Rostock, Germany

**Keywords:** Cancer, Cell biology

## Abstract

Due to the increasing number of human skin cancers and the limited effectiveness of therapies, research into innovative therapeutic approaches is of enormous clinical interest. In recent years, the use of cold atmospheric pressure plasma has become increasingly important as anti-cancer therapy. The combination of plasma with small molecules offers the potential of an effective, tumour-specific, targeted therapy. The synthesised glycosylated and non glycosylated thia-analogous indirubin derivatives KD87 and KD88, respectively, were first to be investigated for their pharmaceutical efficacy in comparison with Indirubin-3'-monoxime (I3M) on human melanoma (A375) and squamous cell carcinoma (A431) cells. In combinatorial studies with plasma-activated medium (PAM) and KD87 we determined significantly decreased cell viability and cell adhesion. Cell cycle analyses revealed a marked G2/M arrest by PAM and a clear apoptotic effect by the glycosylated indirubin derivative KD87 in both cell lines and thus a synergistic anti-cancer effect. I3M had a pro-apoptotic effect only in A431 cells, so we hypothesize a different mode of action of the indirubin derivatives in the two skin cancer cells, possibly due to a different level of the aryl hydrocarbon receptor and an activation of this pathway by nuclear translocation of this receptor and subsequent activation of gene expression.

## Introduction

Skin cancer is one of the most common tumour entities worldwide and is conventionally separated into two categories: melanoma and non-melanoma skin cancer^1^. Malignant melanomas account for only 2–4% of malignant skin cancers overall, but lead to most deaths^2,3^. In contrast, non-melanotic skin cancer accounts for about 25% of all malignant neoplasms diagnosed, while it causes only 0.25% of all cancer deaths^3,4^. The most common types of non-melanotic skin cancer are basal cell carcinoma and squamous cell carcinoma. Squamous cell carcinomas are more likely to invade surrounding tissue and the risk of metastases increases in advanced stages^1,3^. The main goals of skin cancer therapy are the complete removal or destruction of the malignant lesion and an acceptable cosmetic result, which can be achieved by surgical removal or chemo-, immuno- and targeted therapies^5^.

In targeted cancer therapy, antibodies or small molecules can inhibit certain proteins (e.g. mitogen-activated protein kinase) in central signaling pathways for tumour development, growth and metastasis^6^. Unlike antibodies, small molecules can cross the cell membrane and reach intracellular targets. The small molecule indirubin shows anti-cancer effects, but is poorly soluble in water^7^. Indirubin and its analogues are potent ligands for the aryl hydrocarbon receptor (AhR).

The AhR is present in the cytosol in a multiprotein complex composed of hsp90 (heat shock protein of 90 kD), X-associated protein and p23^8^. After ligand binding, the multiprotein complex changes its conformation and is translocated into the nucleus, where the AhR dissociates and binds to the Ah receptor nuclear translocator (Arnt). This heterodimer has a high affinity for binding to the dioxin responsive element, leading to increased transcription of responsive genes, including, for example, the cyclin-dependent kinase 2 (CDK2) inhibitor p27^kip1^^9^. Moreover the activated AhR-complex has been shown to interact with other transcription factors^10^. Recent work has revealed its role in protein degradation as a member of an ubiquitin ligase complex^11^. Additionally, several indirubin derivatives are effective by selectively inhibiting cyclin-dependent kinases (CDK) 1, 2 and 5 and other kinases directly, such as glycogen synthase kinase 3β (GSK-3β), resulting in a subsequent cell cycle arrest^8,12,13^.

In order to further increase the efficacy of indirubin derivatives and to simplify the applicability through improved bioavailability, we and others synthesised novel derivatives^14,15^. The treatment of melanoma-derived cells with thia-analogue indirubin N-glycosides revealed anti-proliferative and pro-apoptotic effects^15^. Based on this promising evidence, we chose a glycosylated (KD87) and a non-glycosylated (KD88) thia-analogous indirubin derivative for our experiments. Since indirubins are normal products in tryptophan metabolism, various derivatives can be detected in serum and urine. Indirubins present in fetal calf serum (~ 0.07 nM) have been found to stimulate AhR signalling in cell culture experiments^16^. For this reason, in vitro experiments with indirubins should be performed under serum-free conditions.

Another innovative treatment option for various cancers is cold atmospheric pressure plasma (CAP). Physical plasma can be defined as an (partially) ionised gas which consists of charged particles (electrons, ions), neutral and electrically excited atoms and molecules, radicals and UV photons^17,18^. When reacting with solutions, the formation of reactive species such as reactive oxygen and nitrogen species (RONS) is facilitated. In plasma-activated fluids, longer-lived reactive species such as H_2_O_2_, NO_2_− and NO_3_− are formed^17,19^. In addition to direct treatment with CAP, indirect treatment with plasma-activated fluids is also possible and shows comparable efficacy^18,20^. The efficacy of plasma-activated cell culture medium (PAM) is attributed to long-lived, stable organic peroxides formed by the reaction of amino acids or proteins with short-lived reactive species^18^. Peroxidised amino acids can react by polymerising to form larger compounds. Due to the long-lived reactive species, the medium activated with plasma continues to exert its effect for up to 21 days after treatment of the medium^21,22^. The effects of CAP have been described in numerous studies; for example, it has a disinfecting effect (bacteria, fungi, viruses), promotes tissue regeneration (pH modulation, angiogenesis), is anti-inflammatory and has an pro-apoptotic and anti-cancer effect^18,19,23,24^. In initial clinical studies with patients with locally advanced squamous cell carcinoma of the oesopharynx, treatment with CAP led to a reduction in odour (antibacterial effect) and a reduced requirement for pain medication. In addition, partial remission of the tumour for at least nine months was observed in two patients^25^. The importance of CAP in combination therapies with chemotherapeutics, e.g. to reduce the dose, increase specificity and circumvent resistance to already applied therapies, has also been investigated^26,27^.

However, the use of PAM and small molecules has not been combined so far. Within the scope of the work, investigations into the combinatorial effect of the novel indirubin derivatives (small molecules) and plasma-activated medium were carried out. We wanted to investigate whether the use of plasma-activated medium leads to a sensitisation of the tumour cells and thus to a synergistic effect with the indirubin derivatives. Our intention was also to consider whether there is a different cellular response to the glycosylated and non-glycosylated indirubin derivatives compared with the established small molecule Indirubin-3'-monoxime (I3M) in combination with plasma therapy.

## Material and methods

### Cell culture

Human cell lines A431 (squamous cell carcinoma) and A375 (melanoma) (both from Cell Line Service) were cultivated at 37 °C and 5% CO_2_ in Dulbecco's Modified Eagle Medium (DMEM; Cat No: 10569; Thermo Fisher Scientific) supplemented with 10% fetal calf serum (FCS; FKS Superior, Biochrom GmbH) and 1% penicillin/streptomycin (P/S; Thermo Fisher Scientific). For passaging, cells were washed twice with Dulbecco's Phosphate Buffered Saline (PBS, Sigma-Aldrich Chemie). The cells were detached with 500 µl of 0.25% trypsin–EDTA (Thermo Fisher Scientific) for A431 and 0.05% trypsin–EDTA (Biochrom GmbH) for A375 and counted with the NucleoCounter NC3000 (ChemoMetec A/S) using a Via1 cassette (ChemoMetec).

### Plasma treatment

The kINPen09 (INP, Leibniz Institute for Plasma Science and Technology) supplied with argon gas (1.9 slm, ALPAHGAZ 1 argon, argon 99.999% purity, AIR LIQUIDE) was used for plasma treatment^28^. By applying a high-frequency voltage (1.1 MHz/2–6 kV) to the needle electrode, a plasma jet is generated^21^. For automated plasma treatment, a 2-axis precision positioning table (Owis GmbH) with the OWISoft software (v. 2.9.1.2) was used. 100 µl of DMEM (w/o FCS) was placed in each well of a 96-well plate.The distance of the capillary to the plate was 1 mm. Dulbecco’s Modified Eagle Medium w/o FCS (**DMEM**) was plasma treated for 30 s per well and this plasma-activated medium (**PAM**) was collected in a reaction vessel and stored for 24 h in the incubator at 37 °C. Argon-activated medium, treated for 30 s only with argon gas (no ignited plasma) served as gas control.

### Small molecules

**Figure 1:**
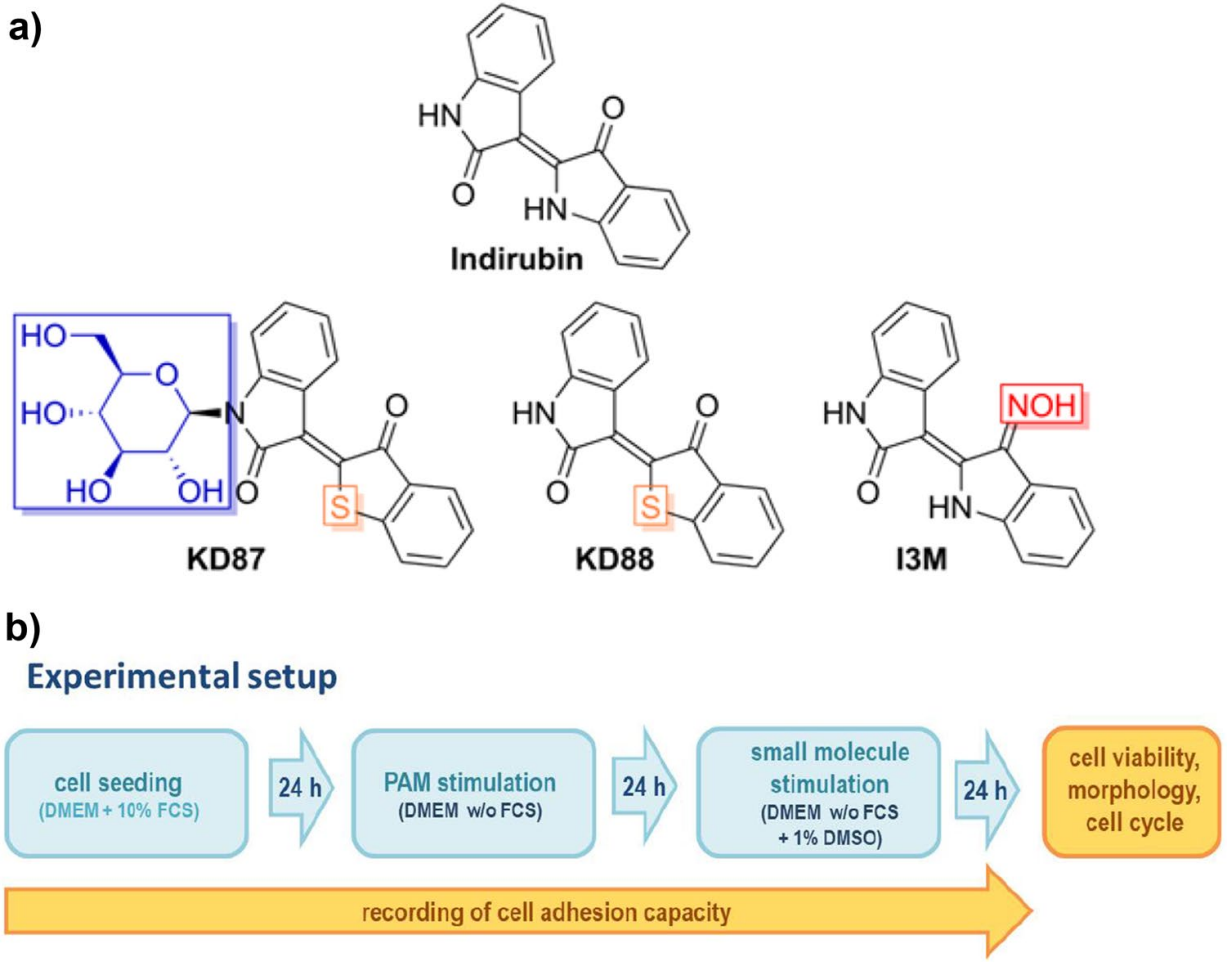
(**a**) Structural formulas of indirubin, thia-analogous indirubin derivatives KD87, KD88 and indirubin-3'-monoxime (I3M). Differences to indirubin are marked in color. (**b**) Overview of the experimental setup to analyse the combinatory effect of plasma treatment (PAM) and small molecule stimulation. All cells were allowed to adhere for 24 h. Cells were first treated with PAM and subsequently with the small molecules.

The indirubin derivatives 3-[Thiaindan-3’-on-2’-(*Z*)-ylidene]-1-(ß-d-glucopyranosyl)indolin-2-one (**KD87**, M = 441.45 g/mol) and (Z)-3-(3-Oxobenzo[b]thiophen-2(3H)-yliden)indolin-2-one (**KD88**, M = 279.31 g/mol) were synthesised starting from isatin (for KD88) and the corresponding glycosylated isatin (for KD87) by condensation with thiaindan-3’-one, respectively (Fig. 1a)^15^. The glycosylated isatin had been prepared in a preceding reaction by cyclisation of the glycosylated aniline with oxalyl chloride following a modified “Stolle”-procedure^29^.

Indirubin-3'-monoxime (I3M, M = 277.28 g/mol) was purchased from Sigma-Aldrich. Stock solutions (10 mM) of all three substances were prepared by dissolving the powder in sterile dimethyl sulphoxide (DMSO; Sigma-Aldrich); these were alliquoted and stored at − 20 °C. DMEM with 1% DMSO served as a vehicle control for all approaches in which cells were stimulated with small molecules. Different dilutions of the indirubin derivatives were tested to determine the most effective concentrations (IC_50_ KD87 for A375 after 24 h: 10 µM). A final concentration of 10 µM was chosen for all derivatives (except for MTS test of A375, 5 µM was measured additionally).

### GSK-3β kinase assay

The indirubin derivatives were tested with the ADP-Glo kinase assay (Promega) for their potential inhibitory effect against GSK-3β enzyme (Promega) according to the supplier’s instructions. The dissolved compounds were diluted in assay buffer and tested in a concentration range of 0.05—100 µM. The final DMSO concentration did not exceed 1%. The GSK-3β kinase reaction was performed in white 96-well plates (Nunc) with 1 ng enzyme, 25 µM ATP and 0.2 µg µl^−1^ GSK-3β substrate with the different test compound concentrations for 60 min at room temperature. Luminescence was recorded after 30 min using a Varioskan Flash Multimode reader (Thermo Scientific, USA). For each test compound, a dose–response curve was measured.

### Combinatory treatment: plasma-activated medium (PAM) and small molecules

The experimental procedure for the combined treatment with PAM and small molecules is shown in Fig. 1b. Due to the reactivity of the plasma-generated reactive species and to exclude an influence of PAM on the conformation of the small molecules, we decided to treat the cells sequentially: first with PAM and then with the small molecules. A375 and A431 cells were seeded in complete medium with 10% FCS and, after a growth phase of 24 h, the cells were washed once with PBS, and then 100 µl PAM or DMEM (all w/o FCS) was added to the cells. After 24 h in the incubator, each medium was aspirated and 100 µl DMEM (w/o FCS) with the small molecules KD87, KD88 or I3M was added to the cells. The detached cells were washed away during the medium change (this was particularly evident with PAM 60 s and PAM 120 s (not shown), which is why PAM 30 s was chosen for all experiments shown in this study). As a control of the small molecule treatment, cells were always incubated with DMEM containing 1% DMSO. After a further 24 h incubation, the combinatorial effects of PAM and small molecules were measured.

### MTS cell viability

The MTS-assay (Cell Titer 96 AQueous One Solution Cell Proliferation Assay, Promega) was performed according to the manufacturers’ instructions. Briefly, the cell number was adjusted to 2 × 10^4^ cells per 96-well, so that the cells in the control group grew into an approximately confluent cell layer in the wells after an overall 72 h duration of the experiment (Fig. 1b). For viablity measurements, 20 µl of MTS reagent was pipetted into each well. Wells without cells were included as blanks (DMEM, PAM, DMEM with 1% DMSO or additionally with small molecules). After 30 min incubation, 80 µl of the supernatant was repipetted from each well into a new 96-well plate. The absorbance was measured on the Anthos-Reader (Anthos Mikrosysteme GmbH) at 492 nm. The amount of MTS converted to formazan is proportional to the number of metabolically active cells. Each blank was subtracted from the respective approach. The measured values for cells in the DMEM control were then normalised to 100%.

### Crystal violet (CV) assay

Cells from the MTS assay were fixed with 100 µl methanol (Avantor) for 10 min, covered with 50 µl Neisser solution II (contains crystal violet and methanol; Carl Roth GmbH) and incubated for 10 min. The amount of the dye that is bound the cells is no longer water-soluble. After washing five times with 150 µl H_2_O, 100 µl of 33% acetic acid (Merck) was added for 10 min to release the dye from the cells. 70 µl of the supernatant was transferred to a new 96-well plate and the photometric measurement was performed on the Anthos reader (620 nm). The absorbance is proportional to the number of adherent cells. The values were normalised to the DMEM control and then offset against the respective values of the MTS measurement. In this way, the cell viability was set in relation to the quantity of adherent cells.

### Cell adhesion capacity

The impedance-based xCELLigence RTCA S16 (ACEA biosciences) platform was used for adhesion measurements. The magnitude of the impedance is dependent on the number, size and shape of the cells. The unitless adhesion capacity is calculated from the impedances at time zero (in the absence of cells) and at the time of measurement. 100 µl complete medium was added to each well of the E-Plate VIEW 16 to determine the blank value. 100 µl of cell suspension (2.5 × 10^4^ cells) was added and the plate was placed in the incubator (37 °C, 5% CO_2_) to start a continuous measurement, with a time interval of 5 min. The values were normalised to the last measured value before the plasma treatment (adhesion capacity = 1.0).

### Cell size

For determination of cell- and nuclei size, the living cells were incubated with Calcein AM (1 µM, Thermo Fisher Scientific) and Hoechst 33342 dye (1 μg/ml, Sigma Aldrich) for 15 min and images were acquired immediately with the LSM 780 (Carl Zeiss AG) at 37 °C. ImageJ (version1.41; NIH) was used to measure the cell size and morphology.

### Aryl hydrocarbon receptor

Adherent cells were fixed with paraformaldehyde (4%, 10 min, Merck KGaA), and permeabilised with Triton X-100 (0.1%, 10 min, Sigma-Aldrich). Staining was performed using a anti-AhR monoclonal antibody (RPT9; 1:50 overnight at 4 °C; Thermo Fisher Scientific) and a goat anti-mouse Alexa 488 secondary antibody (1:100, Thermo Fisher Scientific) and Hoechst 33342 dye (1 μg/ml). Image acquisition was conducted with the LSM 780. The images were recorded with identical settings for quantitative analyses. The profiles were created using the ZEN software (version 2.3).

For AhR-quantification, detached, fixed and AhR-stained cells were measured using quantitative microscopy. Stained cells were inserted into a A2-slide and the fluorescence intensity was measured using the NucleoCounter NC3000 (ChemoMetec). The evaluation of the fluorescence intensities was carried out using the software FlowJo (version 6.2, Becton Dickinson).

### Gene expression analysis

After cultivation for 6 h, total RNA from cells was isolated using the NucleoSpin RNA kit (Macherey-Nagel). First-strand cDNA was synthesized from at least 1 µg total RNA by reverse transcription with SuperScript II (Invitrogen) using 2.5 µM random hexamers (Invitrogen). Quantitative real-time PCR assays were performed using the ABI PRISM 7500 sequence detection system (Applied Biosystems). The PCR reactions contained 8 µl diluted cDNA (fivefold) in a reaction volume of 20 µl, 1 × TaqMan Universal PCR Master Mix (Applied Biosystems) and 1 µl assays-on-demand gene expression assay mix (Applied Biosystems) for the detection of *CYP1A1* (Hs00153120_m1), *CYP1B1* (Hs00164383_m1) for glyceraldehyde 3-phosphate dehydrogenase (*GAPDH*, Hs99999905_m1). PCR amplification was performed in triplicate and repeated in three independent experiments. Relative gene expression values were calculated based on the comparative ΔΔCT-method, normalised to the geometric mean of GAPDH as endogenous controls and calibrated to cells in DMEM.

### Cell cycle and apoptosis

Following treatment, all cells were trypsinised and collected, along with the supernatant containing non-adherent cells. For DNA staining, the 2-step Cell Cycle assay (ChemoMetec) was used. Briefly, the cell number of all samples was adjusted and the cells were resuspended in 50 µl lysis buffer. The cell nuclei were stained with DAPI (10 μg/ml, ChemoMetec). After 5 min, 50 µl stabilisation buffer was added and 10 µl of each cell suspension was measured in the NucleoCounter NC3000. Distribution of the cell cycle was evaluated using FlowJo. Apoptotic cells were determined based on the subG1-peak of the DNA histogram.

### Statistical evaluation

All data are given as a mean ± a standard deviation (SD) or a standard error of the mean (s.e.m.) of at least n = 3 independent experiments, unless otherwise indicated. Statistical analysis was performed using the GraphPad Prism 8 program (GraphPad Software). MTS, CV and cell cycle results were statistically analysed with One-Way Anova (analysis of variance) followed by Tukey's test. Adhesion values were compared using multiple unpaired t-tests. Gene expression results were statistically analysed using Wilcoxon matched-pairs rank test. A significant difference was assumed with a probability of error of p ≤ 0.05 (p value marked with ***p ≤ 0.001; **p ≤ 0.01; *p ≤ 0.05).

## Results

### Combinatory effects of PAM and small molecules on viability of melanoma cells (A375)

The effect of PAM and small molecules on the metabolic activity of the cells was measured via MTS assay.

**Figure 2:**
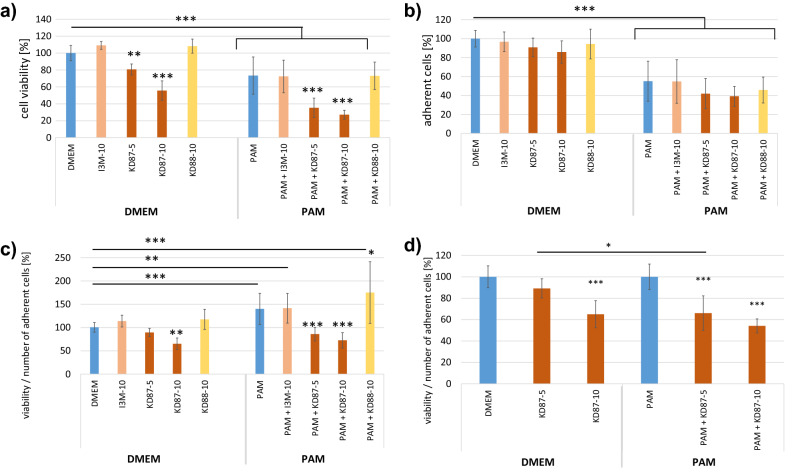
Viability and cell number of A375 cells after the combination of PAM and small molecules. (**a**) Cell viability was significantly reduced by the combination of PAM + KD87-5 (5 µM) and KD87-10 (10 µM). (**b**) PAM led to a significant cell detachment. (**c**) Viability of the remaining adherent cells is shown with mean values normalised to the DMEM control. While PAM increased viability of the remaining adherent cells, treatment with KD87 decreased it. (**d**) The combination of PAM and KD87 5 µM caused a significant loss of viability of the remaining adherent cells, indicating a synergistic effect. The mean values of DMEM and PAM are set 100%. (Tukey's multiple comparisons test, * compared with the respective control (blue columns); n = 3, mean ± SD).

KD87 alone caused a significant reduction in A375 viability of about 35% and 11% at concentrations of 10 µM and 5 µM, respectively (Fig. 2a). Additional treatment with PAM resulted in cell detachment (− 45%) from the surface (Fig. 2b). Interestingly, the remaining cells after PAM showed a higher metabolic activity per cell number (Fig. 2c). It is possible that these cells switched into a “survivor” mode. This was also the case for PAM combined with I3M and KD88. In contrast, PAM + KD87 significantly decreased the viability per number of adherent cells.

The argon controls in combination with the small molecules showed no effect on viability and the number of adherent cells (not shown). The treatment with I3M and KD88 alone caused no effects.

To better illustrate the effect of KD87, the results are set in relation to the respective control (DMEM = 100%; PAM = 100%) in Fig. 2d. A treatment with 10 µM KD87 significantly decreased the viability per number of adherent cells (− 35%). In combination with PAM, both concentrations of KD87 caused a significant decrease (KD87 5 µM: − 34%; 10 µM: − 45.9%) compared with the corresponding PAM control. The small molecule KD87 at a concentration of 5 µM caused a 23% greater loss of viability per number of adherent cells after plasma treatment. This significant difference between the effect of KD87 (5 µM) alone or in combination with PAM indicates a synergistic effect of plasma with the small molecule. However, for KD87 10 µM this synergistic effect is visible, but not significant.

The combination of treatments was also tested in reverse order on the melanoma cell line A375. After 24 h of growth, the small molecules were first added to the cells and after 24 h replaced by PAM. This treatment did not lead to any effect on cell viability. The small molecule treatment showed a viability loss of approx. 35% for KD87 10 µM, independent of the subsequent treatment with PAM.

### Combinatory effects of PAM and small molecules on viability of squamous cell carcinoma (A431)

**Figure 3:**
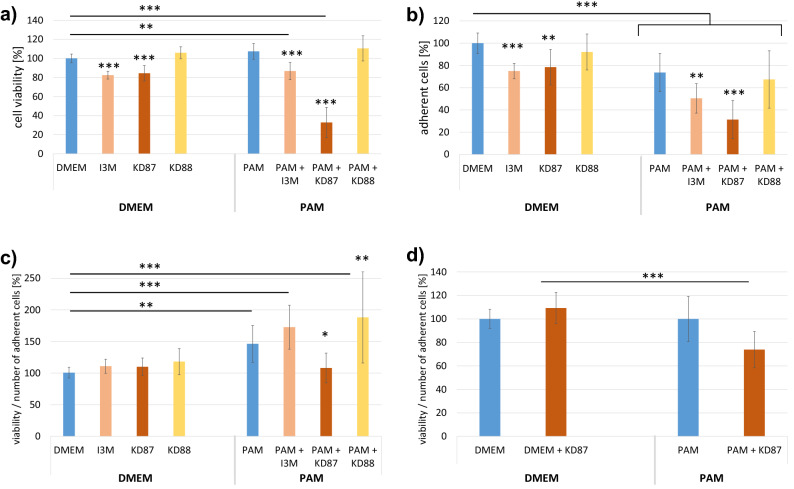
Viability and cell number of A431 cells after combined influence of PAM and small molecules. (**a**) Cell viability was dramatically reduced by the combination of PAM + KD87. (**b**) PAM led to significant cell detachment. (**c**) Viability per number of adherent cells (mean values normalised to the DMEM control). PAM led to a significant increase in viability per number of adherent cells. KD87 counteracted this effect, resulting in values similar to the control. (**d**) The combination of PAM and KD87 caused a significant loss of viability of the remaining adherent cells, indicating a synergistic, anti-cancer effect. The mean values of DMEM and PAM are set 100%. (Indirubin derivatives I3M, KD87, KD88 with 10 µM.Tukey's multiple comparisons test, * compared with the respective control (blue columns); n = 3, mean ± SD).

The viability per number of adherent A431 cells remained unchanged after treatment with either of the small molecules alone **(**Fig. 3c). PAM treatment detached cells from the monolayer by 26% (Fig. 3b), but the remaining, still adherent cells increased their viability by 44% (Fig. 3c). It seems that the remaining cells were stimulated in their metabolic activity due to treatment-induced cell stress. However, treatment with KD87 after PAM stimulation counteracted this response and reduced viability/ adherent cells significantly by 38% (Fig. 3a). This indicates a clear influence of the combination of PAM and KD87 (Fig. 3d).

Treatment with PAM + I3M led to a slight increase of cell viability of about 26% compared with the PAM control. The treatment with KD88 showed an increased cell viability of the adherent cells, with and without previous PAM stimulation. The number of adherent cells was not affected by KD88.

### Cellular adhesion during PAM and small molecule treatment

**Figure 4:**
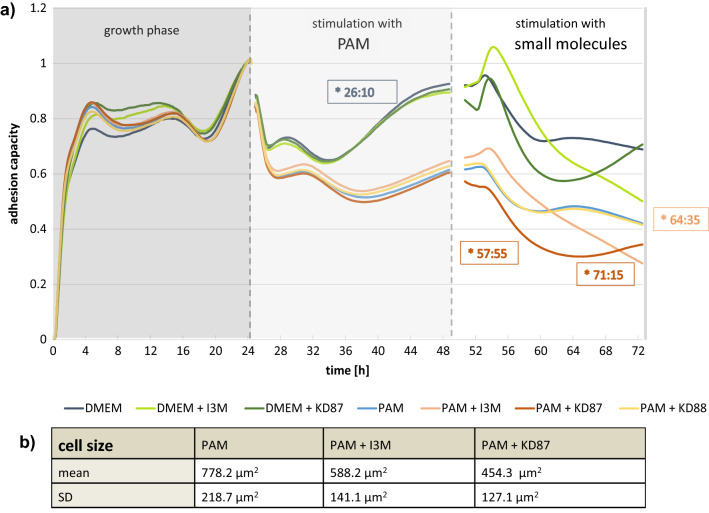
Influence of PAM and small molecules on the time course of A375 cell adhesion based on the impedance measurement. (**a**) Growth phase t0–t24, PAM t24–t48 and subsequent substance incubation t48–t72. The strong decrease in all curves at t24 can be attributed to the change to FCS-free medium (DMEM). The plasma treatment caused a significant decrease in adhesion (from time point 26:10) compared with DMEM (dark blue line). The subsequent stimulation with I3M and KD87 further lowered the adhesion capacity. The time points marked on the right (hh:mm) delimit the area of the adhesion curves that deviates significantly from the DMEM control. (Concentration of all indirubin derivatives was 10 µM; n = 4, mean values normalised to the last value before stimulation with PAM, left dashed line). (**b**) The cell size analysed from the microscopic images (see Fig. S1) after additional small molecule treatment was significantly reduced in comparison to PAM, confirming the reduced adhesion capacity (n = 25 cells).

The adhesion capacity (Fig. 4) of all samples (including the DMEM control) varies throughout the experiment, reflecting normal cell behavior with cell growth, migration, and possibly division. This is why all values were normalised to the time point directly before the first treatment. Immediately after the stimulation at t24, all adhesion curves decreased, presumably due to the switch to serum-free medium. From t26:10 (hh:mm) onwards the adhesion capacity values of the PAM-treated wells were almost consistently significantly lower than those of the DMEM control. At t48 the PAM was replaced and the cells were stimulated with small molecules. Instantly, all adhesion curves dropped, which could be related to the presence of 1% DMSO in all cases.

The curve of the PAM + I3M-treated cells dropped steadily until the end of the measurement, with adhesion capacity values well below those of the PAM control from t64:35, as marked in Fig. 4. The adhesion curve of the PAM + KD87 treatment was significantly lower than the PAM control within the indicated time period. Since the curve increased slightly from t64 onwards, this could indicate a slight recovery of the cells treated with PAM + KD87. Since the impedance can be influenced by several factors (cell size, cell-substrate-attachment, cell–cell-contact) we performed measurements of cell sizes to elucidate which of these parameters might be changed by the small molecules. The cell areas after PAM + I3M and PAM + KD87 treatment were significantly smaller than those of the PAM control (Fig. 4b). The smaller cell areas confirm the lower cell adhesion values measured for I3M and KD87. With regard to cell–cell contacts, we could not detect any difference using the high-resolution images (Fig. S1), so that we can probably exclude an effect of the small molecules on the cell–cell contact of melanoma or squamous cell carcinoma cells.

### Cell cycle and apoptosis rate of A375 and A431

**Figure 5:**
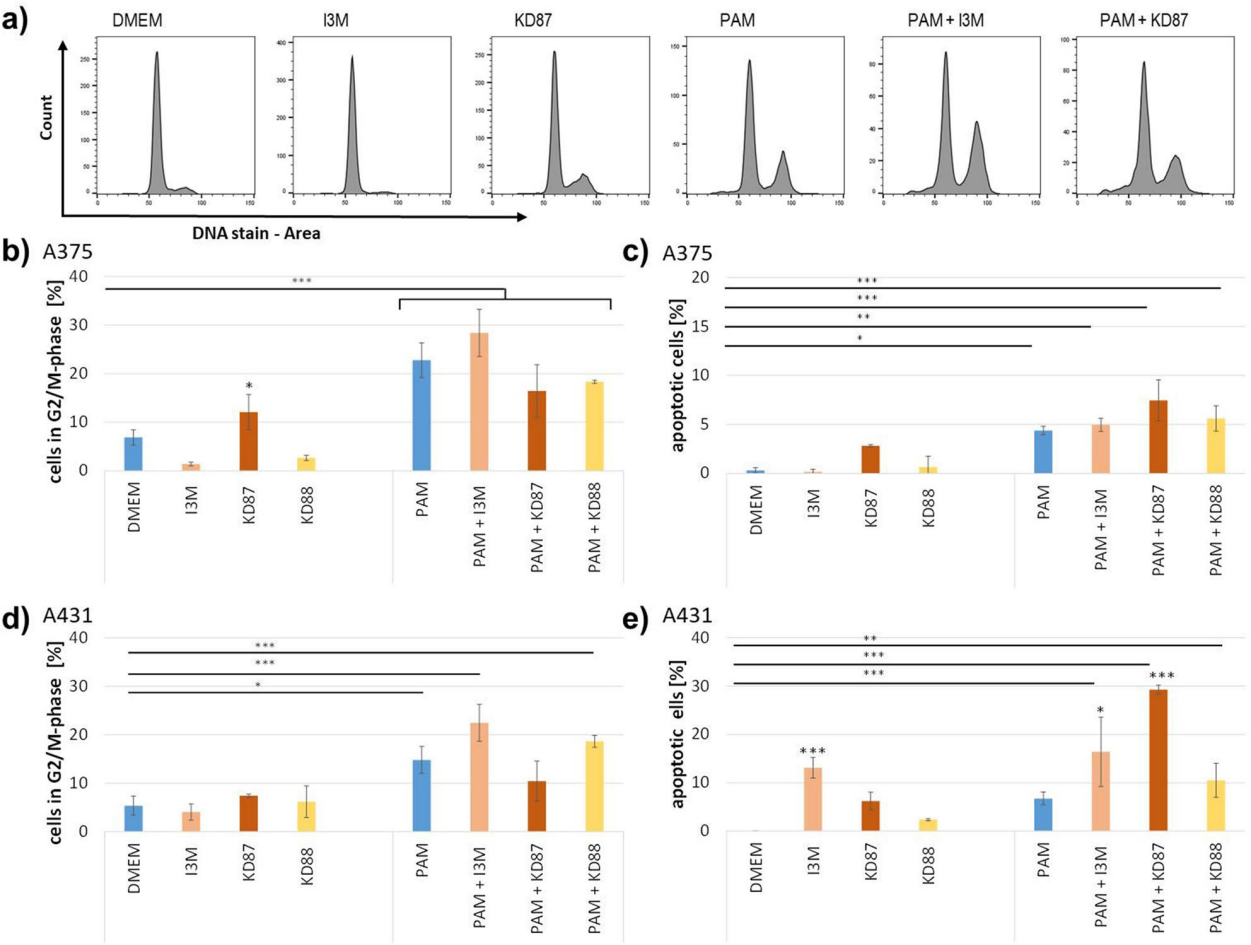
Cell cycle analyses of A375 (**a**–**c**) and A431 (**d**,**e**) cells. (**a**) Exemplary cell cycle histograms of A375 cells. (**b**) Percentage of A375 cells in the G2/M phase. Higher numbers after treatment with PAM indicate an arrest in G2/M, independent of the subsequent small molecule treatment. KD87 alone also caused an increase in cells in G2/M. (**c**) Percentage of apoptotic (subG1) A375 cells rose significantly after PAM. (**d**) The number of cells in the G2/M phase was increased in all PAM-treated A431 cells independent of small molecules. (**e**) Apoptosis was induced in A431 cells by treatment with I3M alone, or by the combination of PAM + either of the small molecules. Concentration of all indirubin derivatives was 10 µM. (ANOVA posthoc Tukey (*) compared with the respective control; n = 3; mean ± SD).

Cell cycle analyses demonstrated that a single treatment with KD87 significantly increased the proportion of A375 cells (Fig. 5) in the G2/M phase (12.1%) compared with DMEM control (6.9%). After I3M, hardly any cells were detected in the G2/M phase. The histogram (Fig. 5a) shows a clear G1 arrest.

The most significant influence, however, was the plasma treatment alone. PAM caused an arrest of the cells in G2/M (22.7%) compared with DMEM (6.9%). This arrest could not be further enhanced or mitigated by subsequent treatment with small molecules. Concerning sub-G1 peak, it can be seen that KD87 led to a slight increase in apoptotic cells. PAM, however, significantly increased the rate of apoptosis (4.4%) compared with DMEM (0.3%). This rate could be further increased by combining PAM + KD87 (7.4%). The pro-apoptotic effect of KD87 could also be confirmed by Annexin V/PI staining (Figs. S2 and S3).

In the case of A431 squamous cell carcinoma cells, there was a clear influence of the indirubin derivatives on the apoptosis rate. The proportion of apoptotic cells was significantly increased by 8.2% with DMEM + I3M and by 4.4% with DMEM + KD87. In combination with PAM, KD87 had a synergistic effect (+ 24.3% compared with DMEM + KD87) on the apoptosis rate. PAM also caused a clear G2/M arrest in these cells, while the single treatment with the small molecules had no effect on the cell cycle. The combination of PAM + I3M clearly increased the arrest of the cells in G2/M (22.5%) compared with PAM (14.8%).

The arrest in G2/M, which is associated with an increased amount of DNA (doubled), could be determined by microscopic analyses (Fig. S1). The size of the nuclei is significantly increased after PAM, indicating a much higher content of DNA.

### Kinase inhibition assay and Aryl hydrocarbon receptor (AhR) expression and translocation

**Figure 6:**
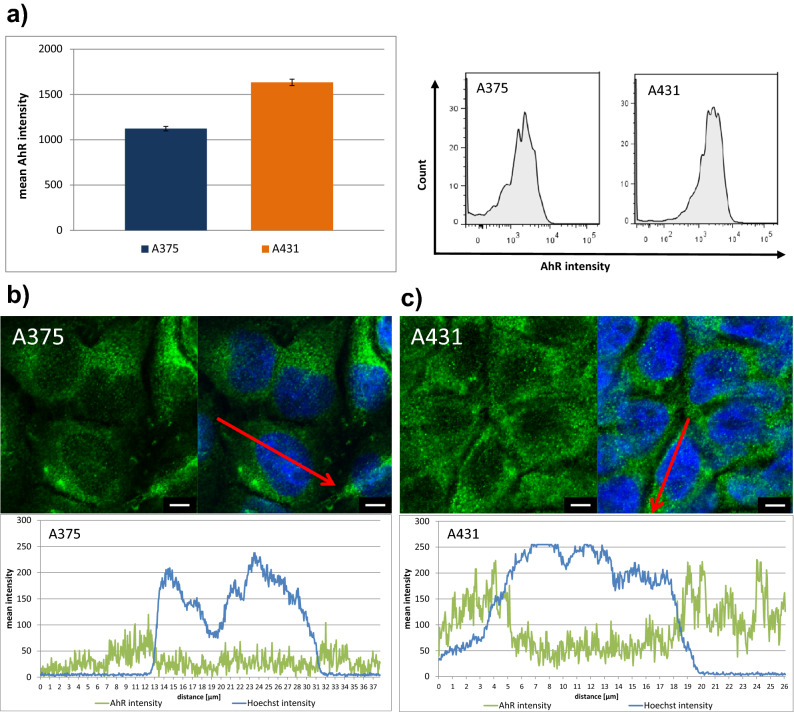
(**a**) Quantification of protein level of the AhR and exemplary histograms demonstrated a tendency to a higher overall expression of AhR in squamous cell carcinoma (A431) cells (NC3000, mean ± SD). Fluorescence images of A375 (**b**) and A431 cells (**c**) showed the localisation of the AhR (green) mostly in the cytosol, with a small fraction being detectable in the nucleus (blue; bar 5 µm). The intensity profile determined along the red arrow is shown underneath the microscopic image. Note the higher basal level of AhR intensity in A431 cells. (confocal microscopy: LSM 780).

To test the mechanism of action of indirubin derivatives, we examined the inhibition of GSK3β kinase and showed that I3M can inhibit this kinase, whereas KD87 and KD88 do not (Fig. 8a). Therefore, we moved on to investigate the AhR pathway. To identify the different modes of action of the indirubin derivatives in the two skin cancer cells, we investigated in a first step whether the expression of the AhR differs. It was determined by quantitative protein analyses (Fig. 6a) that the expression level differs between the cell lines, where A431 tends to have a higher AhR level. This could be confirmed by microscopic images, whereby intensity analyses in cell cross-sections (Fig. 6b,c) showed that the molecule is mostly expressed in the cytosol, with only a small fraction being present in the nucleus.

**Figure 7:**
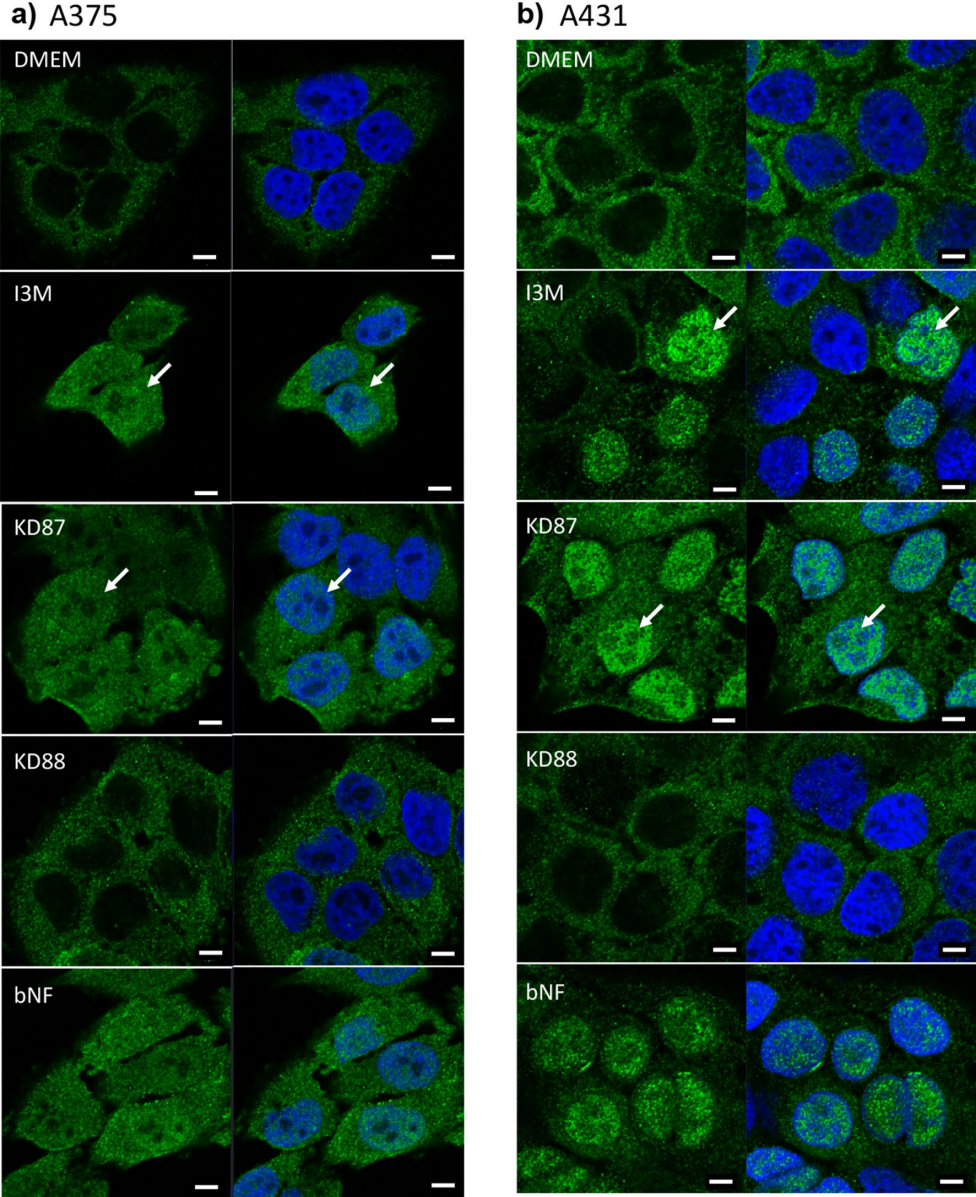
Localisation of the aryl hydrocarbon receptor (AhR, green) after 1 h stimulation with the indirubin derivatives (10 µM). In unstimulated cells (DMEM), the AhR was present in the cytosol. After stimulation with I3M and KD87 the AhR translocated (arrows) to the nucleus (blue). ß-Naphthoflavone (bNF, 20 µM) was used as positive control for AhR-translocation (LSM 780, bars: 5 µm).

To test whether the indirubin derivatives lead to activation of the AhR signaling pathway, the cells were stimulated and translocation of the AhR into the nucleus was monitored (Fig. 7). KD88 did not cause translocation of the AhR receptor, while I3M and the glycosylated KD87 caused a profound accumulation of AhR in the nucleus at this timepoint. This was even more pronounced in A431 cells.

**Figure 8:**
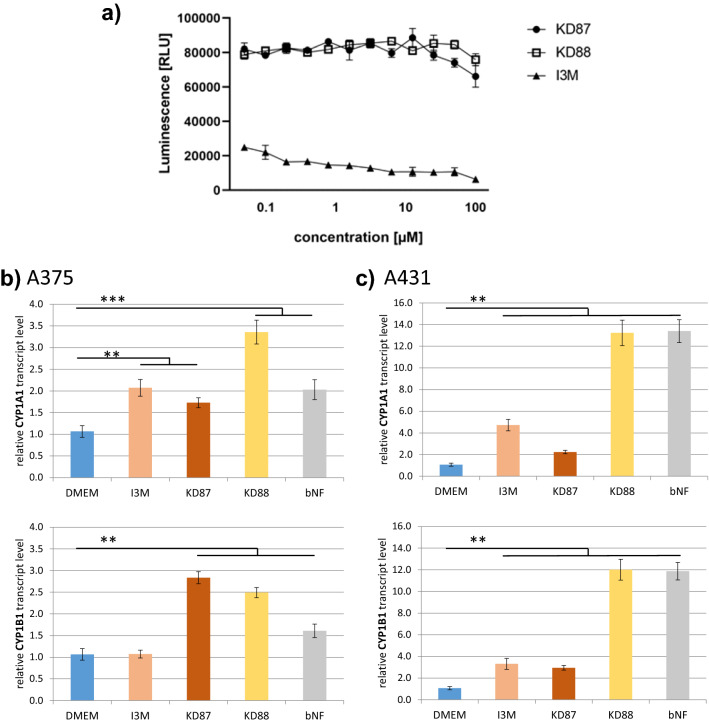
(**a**) Inhibiton of GSK-3β by KD87, KD88 and I3M (Dose–response curves/ADP-Glo kinase assay).Transcript level of the *CYP1A1* and *CYP1B1* genes in A375 (**b**) and A431 (**c**) after 6 h incubation with small molecules. In both cell lines, we observe a significant induction of *CYP1A1* transcript level by KD87, KD88 and bNF. *CYP1B1* transcript levels were significantly increased by KD87 but not by I3M in A375 cells, whereas we found induction by all compounds in A431 cells. ß-Naphthoflavone (bNF, 20 µM) was used as positive control. Note the different scale of the y-axis in (**b**) and (**c**), indicating a more pronounced increase in transcript levels in A431 cells. (*Wilcoxon matched-pairs signed rank test).

Regarding the activation of the AhR pathway we have investigated the markers *CYP1A1* and *CYP1B1* (Fig. 8b,c). We have decided for a stimulation time of 6 h based on the findings of Denison et al.^8^, who have found highest induction of *CYP1A1* transcript level after 6 h stimulation with indirubin. In their case, induction of *CYP1A1* was no longer detectable 24 h after stimulation.

Most prominently, we saw a clear difference in the extent of induction: using ß-naphtoflavone (bNF) as a positive control for AhR activation, we detected a > tenfold increase in transcript level in A431 cells, whereas the maximum induction in A375 was only up to 3.5-fold, which might be related to the higher AhR level in A431 cells. In both cell lines, we found a clear and significant induction of *CYP1A1* transcript level by KD87, KD88 and bNF. *CYP1B1* transcript levels were significantly increased by KD87 but not by I3M in A375 cells, whereas we found induction by all compounds in A431 cells.

## Discussion

Indirubin-3'-monoxime (I3M) is an indirubin derivative that has been widely studied and is considered one of the most potent indirubin derivatives for cancer therapy^30^. I3M acts as a ligand for the aryl hydrocarbon receptor (AhR) and also inhibits several cyclin-dependent kinases (CDK) directly, thereby causing cell cycle arrest. In addition, it has an enhanced anti-proliferative and pro-apoptotic effect. Regarding the metastatic potential of cancer, I3M led to decreased cell migration and invasion^13,30,31^. The inhibitory effect of I3M on tumour proliferation has also been studied in vivo. I3M showed a suppressive effect on tumourigenesis in oral cancer in a mouse model^30^.

Due to the planar structure, pronounced hydrogen bonding and rigid crystal structure, the disadvantage of indirubins is generally their low water solubility^32^. In recent years, different indirubin derivatives were synthesised in order to develop more effective substances for skin cancer therapy^14,15^. Changes in efficacy and water solubility can be achieved by adding additional substituents or exchanging individual atoms. Achieving increased water solubility without loss of bioactivity represents an important goal in the synthesis of new indirubins.

A series of glycosylated thia-analogous indirubin derivatives has been synthesized^15,29,33^ to improve bioavailybility. The substituent of KD87 outlined in blue in Fig. 1 is a carbohydrate residue (ß-D-glucopyranose) which contributes to the significantly improved solubility of KD87. The non-glycosylated analogue KD88 does not carry a carbohydrate substituent and is therefore less soluble. It has been demonstrated that the anti-proliferative activity of various indirubin N-glycosides on human cancer cells in vitro is significantly higher than the activity of the corresponding non-glycosylated derivatives^29^. The oxime (=N–OH) present in I3M (Fig. 1, marked in red) is a derivative of the ketone (=O) found in KD87, KD88 and also in the original indirubin. Since the thia-analogous indirubin compounds (KD87, KD88; orange marking in Fig. 1) are structurally very similar to the original indirubin compounds, testing with regard to their biological activity was worthwhile. N-glycosylated indirubins were tested in another study^29^, where a mostly higher anti-proliferative effect on cancer cells compared with their corresponding non-glycosylated indirubin compounds was shown.

When A375 or A431 cells were pretreated with PAM for 30 s and subsequently treated with KD87, synergistic effects on the metabolic activity and adhesion capacity could be measured. The mechanism by which CAP develops its anti-cancer effect has not yet been sufficiently and comprehensively clarified. Reactive oxygen and nitrogen species (RONS) are thought to play a major role, for example, by exerting oxidative damage to the DNA^17,18,27^. The intracellular formation of ROS also triggers lipid peroxidation reactions, which can lead to damage in the cell membrane and thus to an increased influx of reactive species into the cell, where they influence the metabolism of the cells via the calcium signalling pathway and trigger apoptosis through oxidative stress^18^. The pro-apoptotic effect of cold atmospheric pressure plasma or the fluids treated with it has already been investigated in numerous studies on cancer cells^18,24,34^, including squamous cell carcinoma cells^35^. However, the joint effect of PAM with small molecules has not yet been investigated in more detail.

In the current study, plasma treatment was performed indirectly by generating plasma-activated medium (PAM) that was stored for 24 h and then applied to the cells for stimulation. We have shown in previous work that the pH and osmolality of the medium were unchanged and that storage led to quenching of H_2_O_2_ as well as equilibration of the O_2_ content, so that it could be excluded that these parameters were responsible for the cellular effect. The exact mechanism of action has not yet been elucidated, but the main effect is attributed to long lasting RONS like NO_2_−, NO_3_−, or peroxidised amino acids or proteins contained in the PAM^18,22,36,37^.

Since we observed detachment of cells under the influence of plasma in the current study, it was interesting to monitor cell adhesion over time. Within the first two hours after administration of PAM we observed a strong decrease in cell adhesion. Other authors observed that cancer cells undergo a change in morphology from a spread-out to a contracted form within one hour after plasma treatment. The polarisation of the cells was lost and the cytoplasmatic protrusions were regressed^38^. Moreover, 30 s of plasma treatment (with the same device as in our study) decreased the expression of E-cadherin in keratinocytes^24^. Further treatment with KD87 and also with I3M showed a significant attenuating effect on the adhesion ability. Adhesion values are based on impedance measurements and depend on several parameters, including cell number, cell-substrate or cell–cell adhesion^39^. We determined the cell size after the individual treatments from microscopic images and found a sigificant decrease in cell areas after KD87 and I3M treatment versus PAM control. The lower adhesion could therefore be explained by the reduction in cell size.

It has been postulated that adhesion curves can be defined as "time-dependent profiles of cell response"^40^. These profiles are, on the one hand, cell line specific and on the other hand dependent on the mechanism of action of the substance with which the cells are treated. Thus, a therapy with different substances leads to different adhesion capacity curves depending on the mechanism of action (e.g. CDK inhibition, DNA damage, cell cycle arrest). In contrast, different substances with the same mechanism of action may lead to similar adhesion curves in a cell line.

The influence of indirubins on cell adhesion molecules has not been investigated in detail. However, indirubin promotes the expression of the thrombopoietin receptor, a transmembrane protein, in peripheral blood mononuclear cells^41^. The structure of the cell membrane and extracellular matrix could also be altered via mechanisms that influence cancer cell migration and invasion^31^, with implications for impedance measurement and hence adhesion capacity values.

Another aspect points to the role of the cytosolic AhR in cell migration. The AhR is bound in the cytoplasm to several chaperone proteins and to Src kinase. Activation of AhR leads to rapid release of Src into the cytoplasm, which is accompanied by integrin clustering and subsequent activation of focal adhesion kinase (FAK)^42^. Thus, indirubin molecules activating the AhR could affect FAK activity as well as integrin clustering and regulate cell migration and other processes associated with cell membrane remodeling, thereby changing the cell adhesion capacity, as observed here for I3M and KD87.

In the case of our study, the significantly different adhesion capacity curves after I3M and KD87 treatment thus indicate different mechanisms of action of the small molecules. Therefore, we examined the cell cycle, apoptosis rate and also the AhR-level in more detail.

Regarding its influence on the cell cycle, I3M leads to cell cycle arrest in the G1 or G2/M phase depending on the concentration and the treated cell line^13,43^. Indirubins and their derivatives act intracellularly via binding to the AhR or by inhibiting various cyclin-dependent kinases (CDK) or other kinases (e.g. GSK-3β). Activation of AhR can lead to cell arrest in G1 and G2/M. Thus, downregulation of various cyclins or cyclin-dependent factors, or inhibition of cyclin-dependent kinase inhibitors (such as p21) leads to arrest in G1. In contrast, arrest in G2/M is possible by regulation of CDK1 and M-phase promoting factor^44^. Work by Hoessel et al. showed that I3M exerts its effects more via anti-proliferative and less via proapoptotic effects^13^. We could confirm this, since we found a low apoptosis rate, but a marked G1 arrest after I3M treatment of A375 cells.

However, with the combination treatment, we saw a pronounced effect of PAM on the cell cycle. The G2/M arrest was significant in both cell lines and could not be further enhanced by treatment with the small molecules. Microscopic images also showed a marked increase in nuclear size after PAM, indicating higher DNA content. This marked PAM-induced G2/M arrest has already been described for many cell types, including keratinocytes, and is most likely caused by oxidative damage to the DNA^45^.

The small molecule KD87, which is also described in the work of Kunz et al*.*^15^, already showed an anti-cancer effect in four metastatic melanoma cell lines. The IC50 values of KD87 were below 20 µM in all cell lines. The anti-cancer efficacy of KD87 was confirmed in the present study on another melanoma cell line (A375) and on the squamous cell line (A431). In addition, Kunz et al*.* investigated the effect of glycosylated indirubin derivatives (not including KD87) in more detail, whereby both derivatives led to a significantly increased apoptosis rate and a strong decrease in adherent cells.

Subsequently, the influence of glycosylated indirubin derivatives on the phosphorylation and activation of the cJun and JNK pathways was also investigated by Kunz et al.^15^. Both proteins were less strongly phosphorylated, whereby the phosphorylation of cJun in particular was associated with an influence on cell proliferation.

Using a glycosylated and fluorinated indirubin derivative (DKP-073) Zhivkova et al.^33^ showed that reactive oxygen species (ROS), which were formed intracellularly shortly after the start of treatment (even before the induction of apoptosis), were shown to be the trigger for the apoptosis mechanisms. Although the structure of the small molecule DKP-073 differs significantly from KD87, a similar mode of action would have been possible. However, Berger et al.^46^ could not detect any induction of intracellular ROS after 8- or 24-h incubation with a glycosylated thia analogue indirubin derivative (measured by the H_2_DCFDA assay). With regard to the anticancer effect of KD87, we also did not detect any ROS-dependent difference (Fig. S4), indicating that ROS-mediated apoptosis does not seem to be involved here. These results provided first insights into the mechanisms of action of N-glycosylated indirubins. However, the specific mode of action of KD87 on tumour cells (especially melanoma and squamous cell carcinoma cells) has not yet been sufficiently investigated.

Generally indirubins are known to be ATP-competing inhibitors of several kinases by the formation of hydrogen bonds between the nitrogen group and the ATP binding site of the kinase^47^. Although some related substances with substituents at the amidic nitrogen could be identified which act similarly (GSK-3β inhibition by some *N*-substituted isatins)^48^, this seems not to be the case for KD87 and KD88. Our investigations on GSK-3β show, that from our 3 compounds only I3M inhibits the enzyme.

We therefore assume that the observed cellular effects are probably caused by activation of the AhR pathway. As already mentioned, AhR activation is one main effect, besides CDK inhibition, in the indirubin signaling cascade. Knockaert et al.^49^ described that some indirubin derivatives (I3M and BIO) in mouse hepatomas are more kinase active—leading to stronger cytotoxic effects—while other compounds more strongly activate AhR-dependent signals (MelO, MeBIO), leading to cytostatic effects by inducing G1 cell cycle arrest. However, the action of the various indirubin derivatives and their effect on AhR is highly dependent on the level of functional AhR, and this differs greatly between cells of different tissues^50^.

We determined that the A375 melanoma cells used in this study expressed lower overall levels of AhR than A431 squamous cell carcinoma cells. In these low-AhR melanoma cells, KD87 led to an acivation of the AhR translocation. In high-AhR A431 cells, the KD87 dependent AhR translocation appears to be more pronounced compared to A375 cells after 1 h of stimulation. In both cell lines, we found a clear increase in *CYP1A1* and *CYP1B1* transcript level by the indirubin derivatives. CYP1A1 and CYP1B1 have divergent but overlapping substrate specificities and can produce different metabolites. Recently, several hydroxymetabolites of tryptophan-derived 6-formylindolo[3,2-b]carbazole have been identified that are formed in rat liver^51^. It can be hypothesised that CYP1A1 and CYP1B1 activities produce hydroxy metabolites of indirubin or indirubin derivatives, which in turn cause specific metabolic responses of the cells. Therefore, the differential cellular response of A375 and A431 cells might be related to the differential stimulation of *CYP1A1* and *CYP1B1* genes by the small molecules.

Regarding KD88, we can only speculate why the strong increase of *CYP1A1* and *CYP1B1* transcript level is not reflected in the metabolic analyses of the cells. Adachi et al. describe that the genes induced here are also responsible for the degradation of indirubin molecules^16^. Thus, at least CYP1A1 causes a degradation of indirubin and thus a feedback-regulated autoinhibition of the pathway. In their experiments, an AhR ligand activity of indirubin could no longer be detected in a reporter assay after incubation with CYP1A1 enzyme. Consequently, indirubin is one known substrate of CYP1A1, but further data suggest that also other derivatives of indirubin are endogenous ligands of the AhR and that the ligands are metabolised by the enzymes that they induce^52,53^. It is possible that the extremely strong activation of the *CYP1A1* and the *CYP1B1* genes by KD88 causes a rapid elimination of the substance.

We propose to consider the AhR pathway as one main mechanism of action of glycosylated indirubin derivatives. Thus, the investigations on the AhR-signaling cascade pathways might be promising starting points for the elucidation of the mode of action of glycosylated thia-analogous indirubins like KD87. These underlying mechanisms will be the target of future research.

## Conclusion

Considering the results obtained in this in vitro study, a synergistic effect of plasma-activated medium (PAM) and the novel glycosylated indirubin derivative KD87 on human skin cancer cells could be detected. KD87 alone showed a stronger anti-cancer effect compared with the known small molecule I3M concerning viability, adhesion capacity, apoptosis and G2/M cell cycle arrest, especially in A375 cells. Interestingly, PAM enhanced these effects significantly in skin cancer cells. The non-glycosylated analogue KD88 alone or in combination with PAM showed no effects. PAM combined with KD87 represents a promising, effective therapeutic approach. The mechanism of action of the small molecules could be explained in part by the expression and activation of the aryl hydrocarbon receptor (AhR) pathway in A431 and A375 cells, *e.g.* due to the translocation into the nucleus and the increase in *CYP1A1* and *CYP1B1* transcript level. PAM combined with the small molecule KD87 represents a promising, effective therapeutic approach for dermal cancers.

## Supplementary Information

Supplementary Information.
