# Publisher Correction: Synergistic effect of plasma-activated medium and novel indirubin derivatives on human skin cancer cells by activation of the AhR pathway

**DOI:** 10.1038/s41598-022-08218-9

**Published:** 2022-03-14

**Authors:** Henrike Rebl, Marie Sawade, Martin Hein, Claudia Bergemann, Manuela Wende, Michael Lalk, Peter Langer, Steffen Emmert, Barbara Nebe

**Affiliations:** 1 Department of Cell Biology, Rostock University Medical Center, 18057 Rostock, Germany; 2 Institute for Chemistry, University of Rostock, 18059 Rostock, Germany; 3 Institute for Biochemistry, University of Greifswald, 17487 Greifswald, Germany; 4 Clinic and Polyclinic for Dermatology and Venerology, Rostock University Medical Center, 18057 Rostock, Germany

Correction to: *Scientific Reports* https://doi.org/10.1038/s41598-022-06523-x, published online 15 February 2022

The Funding section in the original version of this Article was omitted. The Funding section now reads:

“We acknowledge the support the European Social Fund (ESF), reference: ESF/14-BM-A55-0002/18, and the Ministry of Education, Science and Culture of Mecklenburg-Vorpommern, Germany for the joint research project ONKOTHER-H.”

The original Article has been corrected.

