# Supplementary material for: Synergistic effect of plasma-activated medium and novel indirubin derivatives on human skin cancer cells by activation of the AhR pathway
Source: Sci Rep. 2022 Feb 15;12:2528. doi: 10.1038/s41598-022-06523-x (PMC8847430; doi:10.1038/s41598-022-06523-x)
Supplement: Supplementary file 1 — Supplementary Information. [file 41598_2022_6523_MOESM1_ESM.docx]

Supplementary Material for

Synergistic effect of plasma-activated medium and novel indirubin derivatives on human skin

cancer cells by activation of the AhR pathway

by

Henrike Rebl, Marie Sawade, Martin Hein, Claudia Bergemann, Manuela Wende, Michael Lalk, Peter Langer, Steffen Emmert, Barbara Nebe

Supplementary Methods

*Annexin V / PI-staining*

Treated cells were detached, washed with PBS and resuspended in 100 µl Annexin V binding buffer (Biotium) containing 2 µl Annexin V CF488A conjugate (Biotium) and 2µl Hoechst 33342 dye (1 μg/ml, Sigma Aldrich) for 15 min at 37°C. After washing twice with PBS, cells were resuspended in 60 µl PI (solution 16, ChemoMetec, 1:50), loaded into a A2-chamber and measured with the NC3000 using the Annexin V protocol. Data evaluation was conducted with the NucleoCounter software.

Supplementary Figures

 *Figure S1: Fluorescence images of living calcein–stained A375 cells (green) and nuclei (blue). The nuclear size was increased after all PAM 30s treatments, confirming the increased DNA content present during G2/M arrest in the cell cycle. (LSM 780; bar 5 µm). Concentration of all indirubin derivatives was 10 µM.*

*Figure S2: Annexin V / PI staining of small molecule treated (a) A375 and (b) A431 cells. Early apoptotic cells (Annexin V-positive) are shown in the lower right quartile. Late apoptotic / necrotic (Annexin V-positive and PI-positive) cells are shown in the upper right quartile. In A375 cells apoptosis was induced only by KD87. In A431 cells I3M and KD87 led to a strong increase in early apoptotic cells. Treatment with 0.01% hydrogen peroxide served as positive control.*

A375 A431

Fig S3: Overview of the Annexin V staining of A375and A431 cells after 24h/ 48h/ 72h incubation with the small molecules (10 µM).

*Figure S4: Viability of A375 cells after small molecule treatment with or without radical scavenger. Treatment with (left) 5mM N-acetyl-cysteine (NAC) or (right) 200mM Trolox did not rescue the cell viability decrease caused by KD87. n=3, mean ± SD.*
